# GATA-3 expression in breast cancer is related to intratumoral M2 macrophage infiltration and tumor differentiation

**DOI:** 10.1371/journal.pone.0283003

**Published:** 2023-03-30

**Authors:** Husam Oda, Elham Hedayati, Annelie Lindström, Ivan Shabo

**Affiliations:** 1 Institution of Medical Biosciences, Clinical Pathology, Umeå University, Umeå, Sweden; 2 Department of Oncology-Pathology, Karolinska Institutet, Stockholm, Sweden; 3 Department of Breast, Endocrine Tumors, and Sarcoma, Karolinska University Hospital, Stockholm, Sweden; 4 Division of Cell Biology, Department of Biomedical and Clinical Sciences, Faculty of Medicine and Health Sciences, Linköping University, Linköping, Sweden; 5 Department of Molecular Medicine and Surgery, Karolinska Institutet, Stockholm, Sweden; Nelson Mandela African Institute of Science and Technology, UNITED REPUBLIC OF TANZANIA

## Abstract

Accumulating evidence indicates that tumor-associated macrophages promote tumor progression and that high macrophage infiltration is correlated with advanced tumor stages and poor prognosis in breast cancer. GATA binding protein 3 (GATA-3) is a differentiation marker related to differentiated states in breast cancer. In this study, we explore how the extent of MI relates to GATA-3 expression, hormonal status, and the differentiation grade of breast cancer. To examine breast cancer in early development, we selected 83 patients that were treated with radical breast-conserving surgery (R0), without lymph node metastases (N0) or distant metastases (M0), with and without postoperative radiotherapy. Immunostaining of M2-macrophage-specific antigen CD163 was used to detect tumor-associated macrophages, and macrophage infiltration was estimated semi-quantitatively into no/low, moderate, and high infiltration. The macrophage infiltration was compared to GATA-3, estrogen receptor (ER), progesterone receptor (PR), human epidermal growth factor receptor 2 (HER-2), and Ki-67 expression in cancer cells. GATA-3 expression is associated with ER and PR expression but inversely correlated to macrophage infiltration and Nottingham histologic grade. High macrophage infiltration in advanced tumor grade was associated with low GATA-3 expression. The disease-free survival is inversely related to Nottingham histologic grade in patients having tumors with no/low macrophage infiltration, a difference that is not found in patients with moderate/high macrophage infiltration. These findings indicate that macrophage infiltration might impact the differentiation, malignant behavior, and prognosis of breast cancer, regardless of the morphological and hormonal states of the cancer cells in the primary tumor.

## Introduction

Differentiation is inversely correlated to malignancy and the metastatic potential of solid tumors. Neoplasia with a high degree of differentiation morphologically resembles their native tissue, whereas those with low differentiation lose their structural organization with reduced cohesiveness. Tumor growth and invasion are complicated multifactorial, but debated mechanisms involving interaction between cancer cells and the host tissue microenvironment [1]. Many solid tumors exhibit phenotypic heterogeneity, consisting of multiclonal tumor cells with different functional states. Besides tumor cells, solid tumors are comprised of stroma cells, such as infiltrating immune cells, which collectively determine the malignant behavior of cancer. Moreover, heterogeneity in cancer cell states and tumor microenvironment can possess drug sensitivities, resulting in challenges for therapeutic and prognostic assessment of solid tumors [1, 2].

Breast cancer (BC) arises from multipotent BC progenitor or stem cells yielding various tumor morphologies [3]. Nottingham histologic grade (NHG) is a scoring system used to assess the morphological differentiation in BC. It is based on tumor growth pattern and degree of differentiation, reflecting the similarities of the BC to normal breast epithelial cells. GATA binding protein 3 (GATA-3) is a transcription factor involved in critical steps of differentiation and morphogenesis of several cell types, including mammary epithelial cells, T-helper cells, and nephritic ductal cells [4]. In mammary tissue, GATA-3 is associated with estrogen receptor (ER) expression in the luminal epithelial cells and is highly expressed in well-differentiated BC [5]. The loss of GATA-3 expression results in decreased ER expression and decreases proportionally the more the BC loses its luminal differentiation. Moreover, higher expression of GATA-3 predicts a better BC prognosis and response for hormonal treatment [6, 7].

Macrophages are a heterogeneous population of innate immune cells originating from blood monocytes. In response to different stimuli in the tissue microenvironment, macrophages exhibit two polarization states, M1 and M2. The M1 macrophages are pro-inflammatory and possess microbicidal/tumoricidal activity. The M2 macrophages have an immunosuppressive phenotype, release anti-inflammatory cytokines, and enhance angiogenesis and tissue repair. Tumor-associated macrophages (TAMs) represent the M2 phenotype and constitute a main component of the microenvironment in several solid tumors, enhancing tumor progression, angiogenesis, and chemoresistance [8–11]. In BC, high macrophage infiltration (MI) is associated with poor prognostic phenotype, such as high histological grade, low ER status, and increased Ki-67 proliferation index [12, 13]. In a previous study, we reported significant differences in MI among the intrinsic subtypes of pathologic pT1-T2 BC. The ER expression by BC cells was inversely related to MI [14].

Understanding BC phenotypic differentiation is essential for histological classification and facilitates accurate disease behaviour, prognosis, and management prediction. In this study, we investigate the expression of GATA-3, as a mammary differentiation marker, in relation to M2 macrophage infiltration and differentiation estimated as NHG in BC.

## Materials and methods

### Patient material and study design

We retrospectively obtained data on all patients (n = 1164) with BC with isolated ipsilateral local recurrence (ILR) during 1983–2008 from the breast cancer registry of the south-eastern region of Sweden. For comparison, we selected an age-matched patient cohort (n = 1164) treated during the same period and without ILR. To examine a homogeneous group of BCs in early development, only patients with radically removed tumors (R0), without lymph node metastases (N0), or distant metastases (M0) were selected. This retrospective design enabled including patients not offered postoperative radiotherapy, as it was fully implemented in clinical routine in the early 1990s [15]. To explore possible associations between radiotherapy, MI, functional and phenotypic differentiation, and GATA-3 expression in BC tissue, 50% of selected cases were treated with postoperative radiotherapy (RT). A detailed description of the entire patient group and the selection procedure is described in a flow chart attached as a supplement document S1 Fig. Tumor histology was reviewed by an experienced pathologist (SG), and formalin-fixed paraffin-embedded tissue blocks with invasive BC were chosen for tissue microarray and constructed using two tissue cores (diameter 0.6 mm). Eighty-three patient samples were included in the total. Ethical approval from the Regional Ethics Committee in Linköping was obtained according to Swedish Biobank Law (reference number: 2010/311–31).

### Immunostaining and evaluation

Five-micrometer sections were obtained from formalin-fixed paraffin-embedded TMA tumor specimens. The sections were de-paraffinized in xylene and hydrated in a series of graded alcohols, pre-treated with Heat Induced Epitope Retrieval and TrisEthylenediaminetetraacetic acid (EDTA) buffer (1 mM, pH 9, 20/5/20 min; Decloaking Chamber NxGen, Biocare Medical), and stained for M2 macrophage-specific marker CD163 (anti-human, monoclonal antibody, clone 10D6, Novocastra, Leica) and GATA-3 (anti-human, monoclonal antibody, clone L50-823, ThermoFisher Scientific). Staining for estrogen receptor (ER; clone SP1, Ventana Roche), progesterone receptor (PR; clone 1E2, Ventana Roche), Ki-67 (clone MIB-1, Dako Agilent), and human epidermal growth factor receptor 2 (HER2; clone 4B5, Ventana Roche) was done according to clinical laboratory standards. All slides were scanned to digital images using the Hamamatsu NanoZoomer XL (Visiopharm LRI AB). Evaluation of immunostaining was performed by ImageScope viewing software (Leica Biosystems).

**Fig 1:**
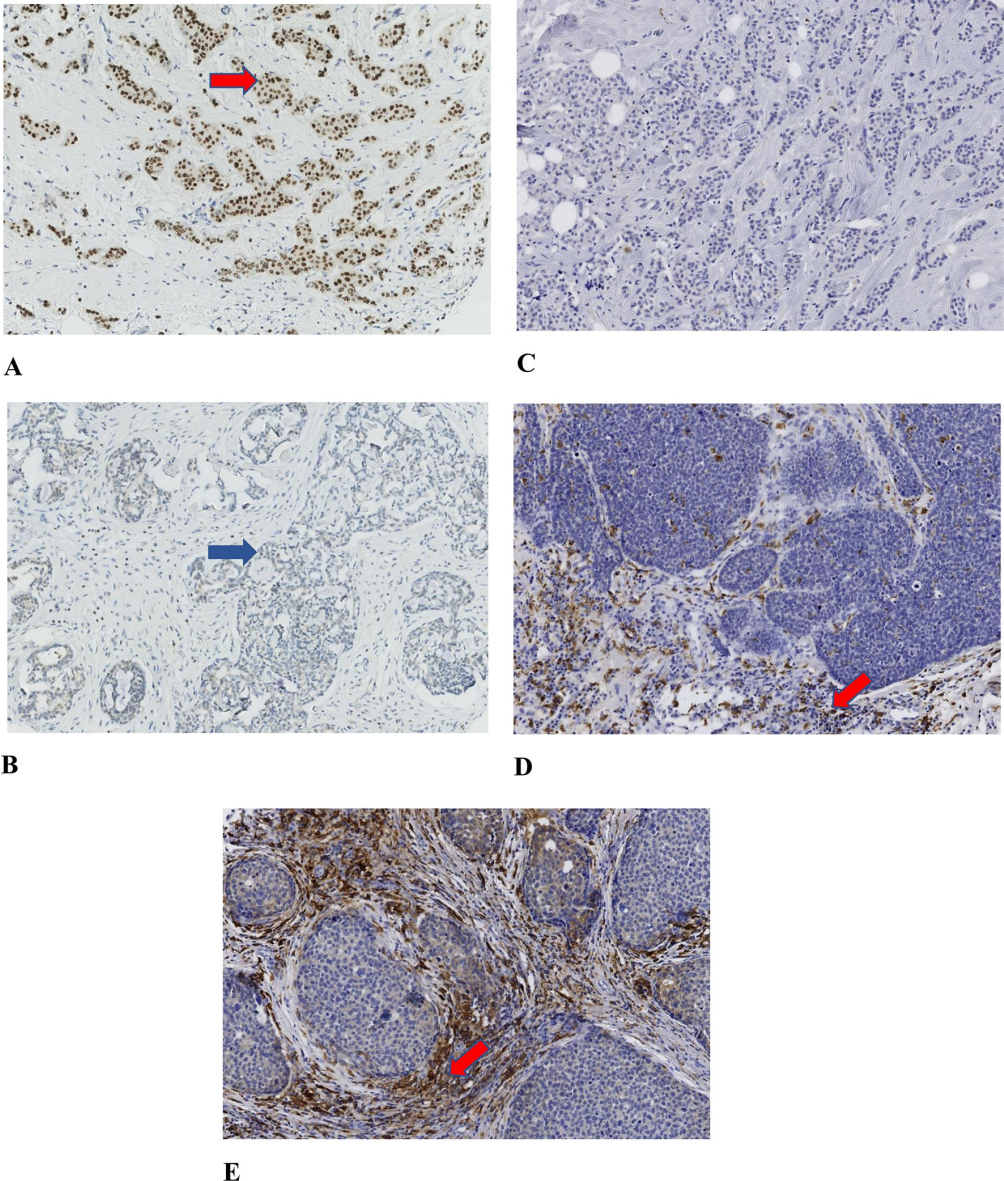
Breast cancer immunohistochemistry images. Image A shows a tumor with GATA-3 positive cancer cells (nuclear staining marked with a red arrow). Image B illustrates a GATA-3 negative tumor where cell nuclei (blue arrow) show no GATA-3 staining. Images C-E show immunohistochemistry for M2-macrophage specific marker CD163 used for detecting M2-macrophages (red arrow) and scoring macrophage infiltration. Images C, D, and E illustrate No/low, moderate, and high macrophage infiltration, respectively.

Two experienced pathologists (SG and HO) evaluated all immunostaining, blinded to patient characteristics and outcomes. The fraction of GATA-3-positive cancer cells was calculated based on a count of 200 tumor cells in each TMA core. TAM infiltration was evaluated semi-quantitatively into three grades: no/low, moderate, or high (Fig 1). The expression of Ki-67, ER, PR, and HER-2 in cancer cells was evaluated according to ESMO guidelines (2019) [16].

### Statistical analysis

SPSS statistics software, version 28 (IBM Corporation, USA), was used for the statistical analyses. MI was evaluated in relation to clinicopathologic data using Pearson´s chi-square test. For continuous data, one-way analysis of variance (ANOVA) was used with a post-hoc Bonferroni’s test for comparing the means between several than two variables. Mann-Whitney test for comparing the means between two variables. Survival rates were estimated according to Kaplan Meier based on disease-free survival (DFS). The statistical significance of differences between survival rates was determined by the log-rank test. For all analyses, p <0.05 (double-sided) was considered statistically significant. A heat map, created by excel software (Office 365), was used to assess the multidimensional association of NHG with MI and proportion rates of cancer cells expressing GATA-3, ER, PR and Ki-67.

## Results

### GATA-3 expression

**Fig 2:**
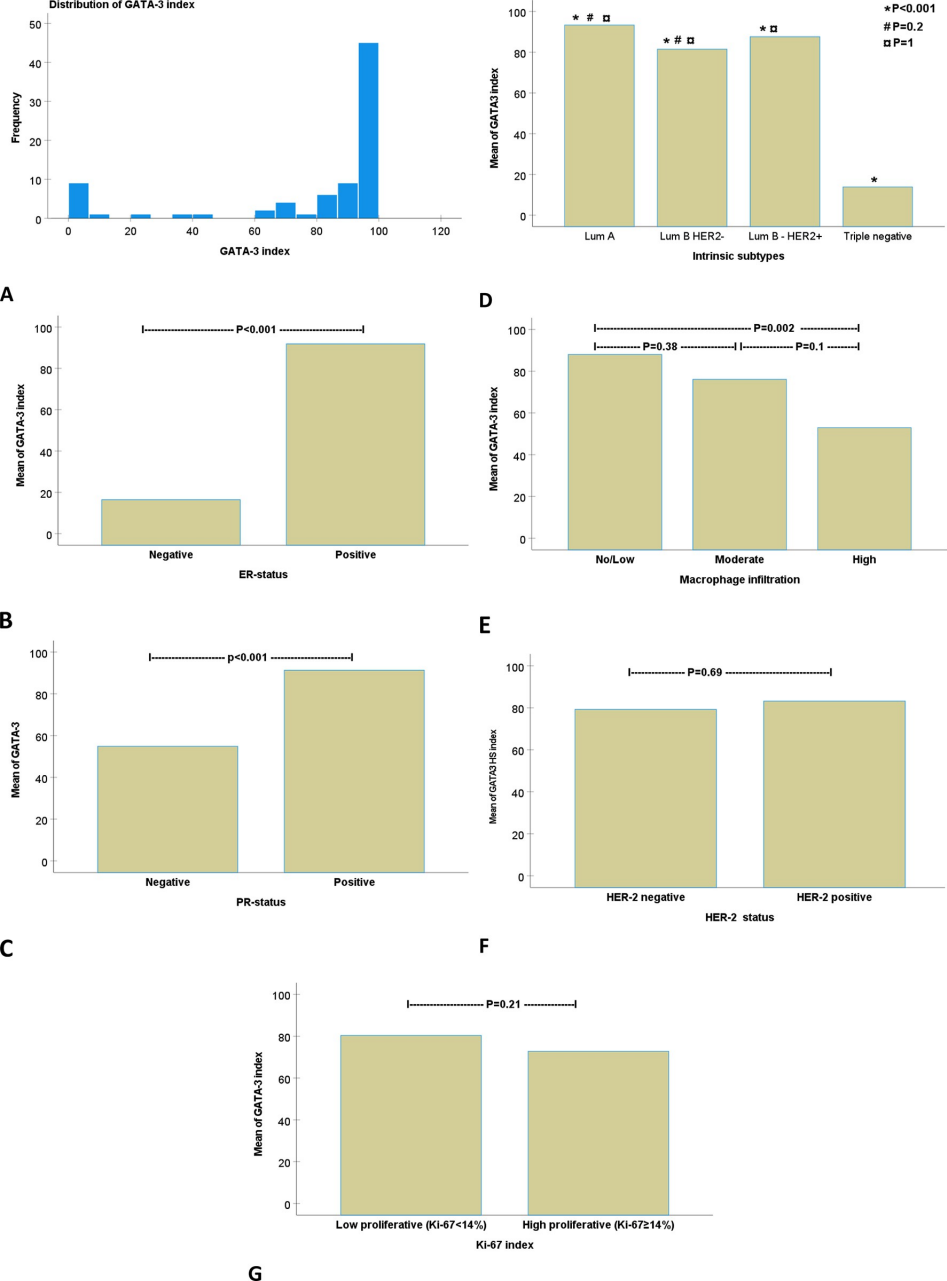
The relation of GATA-3 indexes to macrophage infiltration and functional differentiation of breast cancer. Panel A illustrates the distribution of GATA- 3 indexes. Panels B-G show comparisons of the GATA-3 index in relation to ER status, PR status, intrinsic subtypes, macrophage infiltration, HER-2 status, and Ki-67 index, respectively. For continuous data, one-way analysis of variance (ANOVA) was used with a post-hoc Bonferroni’s test for comparing the means between several than two variables. Mann-Whitney test for comparing the means between two variables.

**Fig 3:**
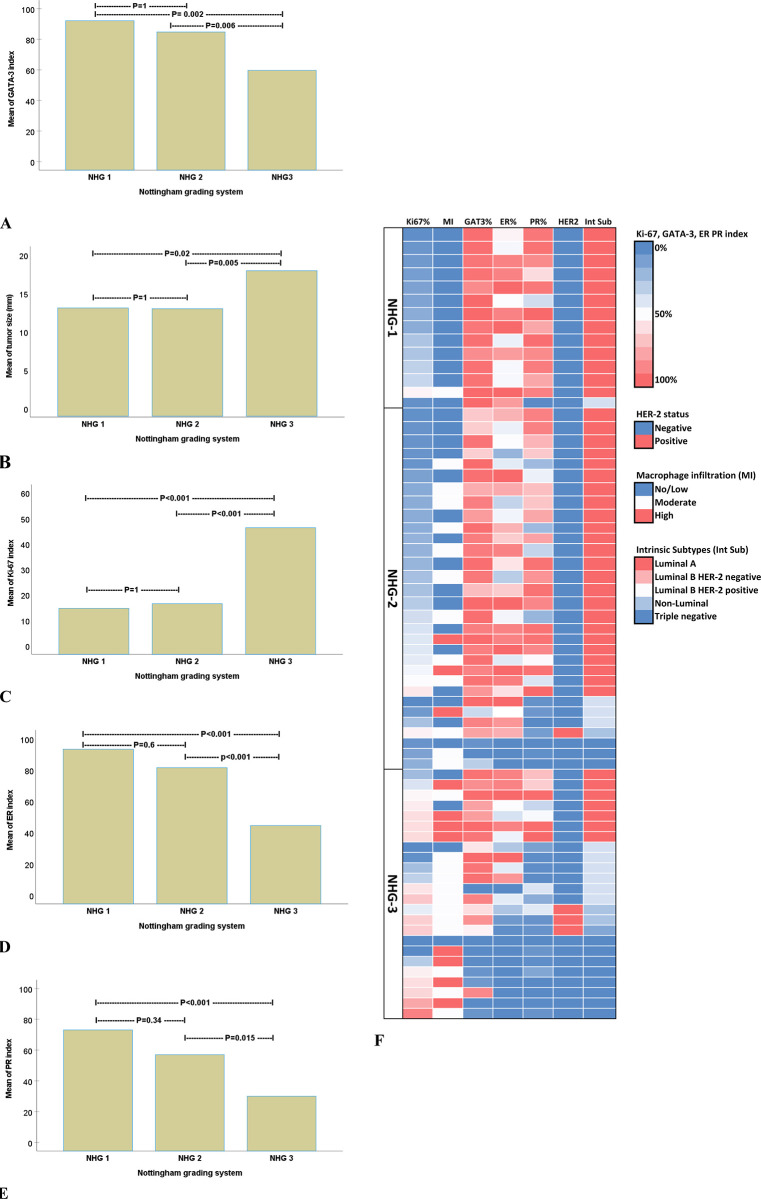
ANOVA analysis followed by Bonferroni’s post hoc test comparing breast cancer Nottingham Histologic Grade (NHG) in relation to (A) GATA-3 index, (B) tumor size, (C) Ki-67 index, (D) ER index and (E) PR index. (F) Heat map of breast cancer NHG visualizes how segregated the distribution of macrophage infiltration, intrinsic subtypes, HER2 status, GATA3, ER, and PR indexes are between the NHG1, NHG2, and NHG3 tumors, respectively.

In total, 83 patients were included in this study. Patient characteristics are summarised in Table 1. GATA-3 was expressed in 78 (94%) cases, of which 67 (84%) patients had tumors where GATA-3 expression was present in >60% of tumor cells (Fig 2A). No difference in GATA-3 expression was found between NHG1 and NHG2, with GATA-3 mean expression rates of 92% (±SD 17.5) and 85% (±SD 25), respectively. The mean GATA-3 index in NHG3 tumors was 60% (±SD 42), which was significantly lower compared to NHG1 (p = 0.002) and NHG2 (p = 0.006) tumors (Fig 3A). GATA-3 expression was significantly lower in ER-negative (mean 16%, ±SD 29) compared to ER-positive (mean 92%, ±SD 12) tumors (p<0.001). The corresponding rates in PR negative vs. positive tumors were 55% (±SD 43) and 91% (±SD 15), respectively (p<0.001) (Fig 2B and 2C). GATA-3 expression in triple-negative tumors (mean 14%, ±SD 27) was significantly less than in Luminal A (mean 93%, ±SD 9), Luminal B HER-2 negative (mean 81%, ±SD 31) and Luminal B HER-2 positive (mean 87%, ±SD 13) tumors (p<0.001). No differences in GATA-3 expression were found between luminal A and B tumors (Fig 2D).

**Table 1:** Patient characteristics.

Variables	N (%)
**Age groups (years)**	
≤40	15 (18)
41–50	18 (22)
51–60	17 (20)
61–70	15 (18)
≥70	18 (22)
**Pathologic T-stage**	
pT1	70 (84)
pT2	13 (16)
**Nottingham grade**	
NHG 1	20 (24)
NHG 2	38 (46)
NHG 3	25 (30)
**ER-status**	
Negative	14 (21)
Positive	66 (79)
Missing data	3
**PR-status**	
Negative	27 (33)
Positive	55 (67)
Missing data	1
**HER2-status**	
Negative	73 (92)
Positive	6 (8)
Missing data	4
**Ki-67-expression**	
<14%	45 (63)
≥14%	27 (37)
Missing data	11
**Postoperative radiotherapy**	
No	42 (51)
Yes	41 (49)
**Local recurrence**	
No	44 (53)
Yes	39 (47)
**Macrophage infiltration**	
No/Low	41 (50.6)
Moderate	28 (34.6)
High	12 (14.8)
Missing data	2
**Intrinsic subtypes**	
Luminal A	49 (62.8)
Luminal B HER-2 negative	12 (15.4)
Luminal B HER-2 positive	5 (6.4)
Non-Luminal	1 (1.3)
Triple-negative	11 (14.1)

Abbreviations: Estrogen receptor (ER), progesterone receptor (PR), human epidermal growth factor receptor 2 (HER-2).

GATA-3 expression was inversely correlated to MI. The mean index of GATA-3 in tumors with No/Low, moderate, and high MI were 88% (±SD 22), 76% (±SD 35), and 53% (±SD 44), respectively. The expression of GATA-3 in tumors with high MI was significantly lower than in tumors with no/low MI (p = 0.002). The differences in GATA-3 expression were not statistically significant comparing tumors with high vs. moderate (p = 0.1) MI and no/low vs. moderate (p = 0.38) MI (Fig 2E). No differences in GATA-3 expression were found in relation to the expression of Ki-67 nor HER-2 status (Fig 2F and 2G).

### NHG in relation to hormonal status

NHG1 and NHG2 tumors had similar sizes (mean 13 mm) and were significantly smaller than NHG3 tumors (mean 18 mm). There were no differences in Ki-67 index between NHG1 (15%) and NHG2 (16.5%) tumors (p = 1), whereas NHG3 tumors exhibited higher Ki-67 index (46%) compared to NHG1 (p<0.001) and NHG2 (p<0.001) tumors (Fig 3B and 3C).

The expression rates of ER were significantly lower in NHG3 tumors (mean index 45%) compared to NHG1 (mean index 93%, p<0001) and NHG2 (mean index 62%, p<0.001). The corresponding rates for PR expression in NHG1, NHG2, and NHG3 tumors were 73%, 57%, and 30%. PR expression in NHG3 tumors is significantly lower than in NHG1 (p<0.001) and NHG2 (p = 0.015) tumors. No differences in ER and PR expression were found between NHG1 and NHG2 tumors (Fig 3D and 3E). HER-2 was almost exclusively expressed in NHG3 tumors. HER-2 expression was found in only one case with an NHG2 tumor. No HER-2 expression was found in NHG1 tumors.

The heat map shows that ER and PR decrease gradually in relation to NHG, of which the lowest ER and PR index rates occur in NHG 3 tumors. However, low ER and PR indices occur also in NHG1 and NHG2 tumors. The intrinsic subtypes among NHG 1–3 tumors were distinctly distributed, where NHG1 and NHG2 tumors had virtually exclusively luminal phenotypes, while NHG3 tumors exhibited non-luminal and triple-negative phenotypes (Fig 3F).

### NHG in relation to MI and Ki-67 index

Macrophage infiltration was inversely correlated to the differentiation grade of BC. Out of 14 tumors with high MI, 8 (67%) had NHG3. The corresponding rates for tumors with no/low and moderate MI were 4 out of 41 (10%) and 13 out of 28 (46%), respectively. Inversely, NHG1 and NHG2 were found in 17 (41%) and 20 (49%) tumors with no/low MI (p<0.001) (Table 2).

**Table 2:** Univariate analysis comparing Nottingham Histologic Grade (NHG) in relation to macrophage infiltration and clinical data in breast cancer.

		Nottingham grade			
	NHG1		NHG2	NHG3	p
	n (%)		n (%)	n (%)	
**Age groups (years)**					
≤40	4 (20)		4 (10.5)	7 (28)	
41–50	3 (15)		9 (24)	6 (24)	
51–60	8 (30)		10 (26)	1 (4)	
61–70	5 (25)		9 (24)	1 (4)	
≥70	2 (10)		6 (16)	10 (40)	0.024
**Pathologic T-stage**					
pT1	19 (95)		34 (90)	17 (68)	
pT2	1 (5)		4 (10)	8 (32)	0.023
**Macrophage infiltration**					
No/Low	17 (85)		20 (56)	4 (16)	
Moderate	2 (10)		13 (36)	13 (52)	
High	1 (5)		3 (8)	8 (32)	<0.001
**ER-status**					
Negative	0 (0)		3 (9)	11 (44)	
Positive	20 (100)		32 (91)	14 (56)	<0.001
**PR-status**					
Negative	3 (15)		9 (24)	15 (60)	
Positive	17 (85)		28 (76)	10 (40)	0.002
**HER2-status**					
Negative	18 (100)		34 (92)	21 (88)	
Positive	0 (0)		3 (8)	3 (12)	0.3
**Ki-67 index**					
<14%	13 (87)		24 (75)	8 (32)	
≥14%	2 (13)		8 (25)	17 (68)	<0.001
**Local recurrence**					
No	12 (60)		21 (55)	11 (44)	
Yes	8 (40)		17 (45)	14 (56)	0.5
**Intrinsic Subtypes**					
Luminal A	16 (89)		26 (72.2)	7 (29.2)	
Luminal B Her-2 negative	2 (11)		4 (11.1)	6 (25)	
Luminal B Her-2 positive	0 (0)		3 (8.3)	2 (8.3)	
Non-Luminal	0 (0)		0 (0)	1 (4.2)	
Triple-negative	0 (0)		3 (8.3)	8 (33.3)	0.004

Abbreviations: Estrogen receptor (ER), progesterone receptor (PR), human epidermal growth factor receptor 2 (HER-2).

NHG3 was more common (17 out of 27, 63%) in high proliferative tumors compared to those with low proliferative tumors (Ki-67<14%) (8 out of 45, 18%). The corresponding rates for high and low proliferative tumors in relation to NHG1 are 2 (7%) and (29%), respectively (p<0.001). As was known before, NHG was related to age (p = 0.024), T-stage (p = 0.023), and intrinsic subtypes (p = 0.004) (Table 2).

### Macrophage infiltration in relation to Ki-67, ER, and PR status

**Fig 4:**
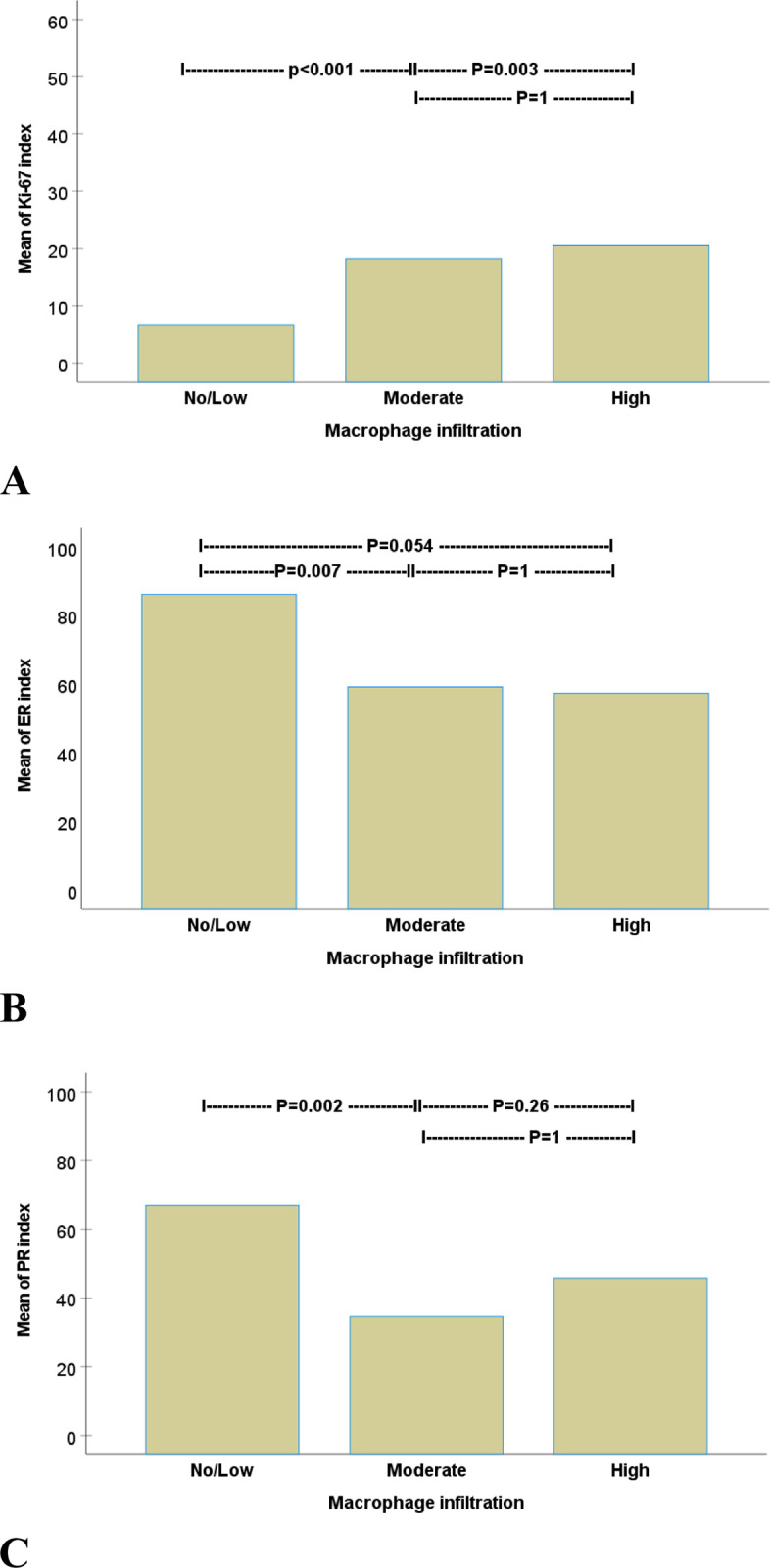
ANOVA analysis followed by Bonferroni’s post hoc test comparing macrophage infiltration in breast cancer in relation to (A) Ki-67, (B) ER, and (C) PR indexes.

The Ki-67 index was proportional to MI, with a mean index of 6.6%, 18%, and 20.6% in no/low, moderate, and high MI, respectively. Ki-67 was significantly lower in tumors with no/low MI compared to those with moderate (p<0.001) and high (p = 0.003) MI. There was no difference in the Ki-67 index between tumors with moderate and high MI (Fig 4A).

Tumors with no/low MI exhibited a mean ER index of 86%, higher than tumors with moderate (59%, P = 0.007) and high (57%, p = 0.054). The corresponding rates for PR index in tumors with no/low, moderate, and high MI were 66%, 35%, and 46%, respectively. The PR was significantly higher in tumors with no/low compared to moderate MI (p = 0.002). No differences in PR index were found between tumors with moderate and high MI (p = 1) nor between no/low and high MI (p = 0.26) (Fig 4B and 4C).

### The prognostic impact of MI in relation to NHG

**Fig 5:**
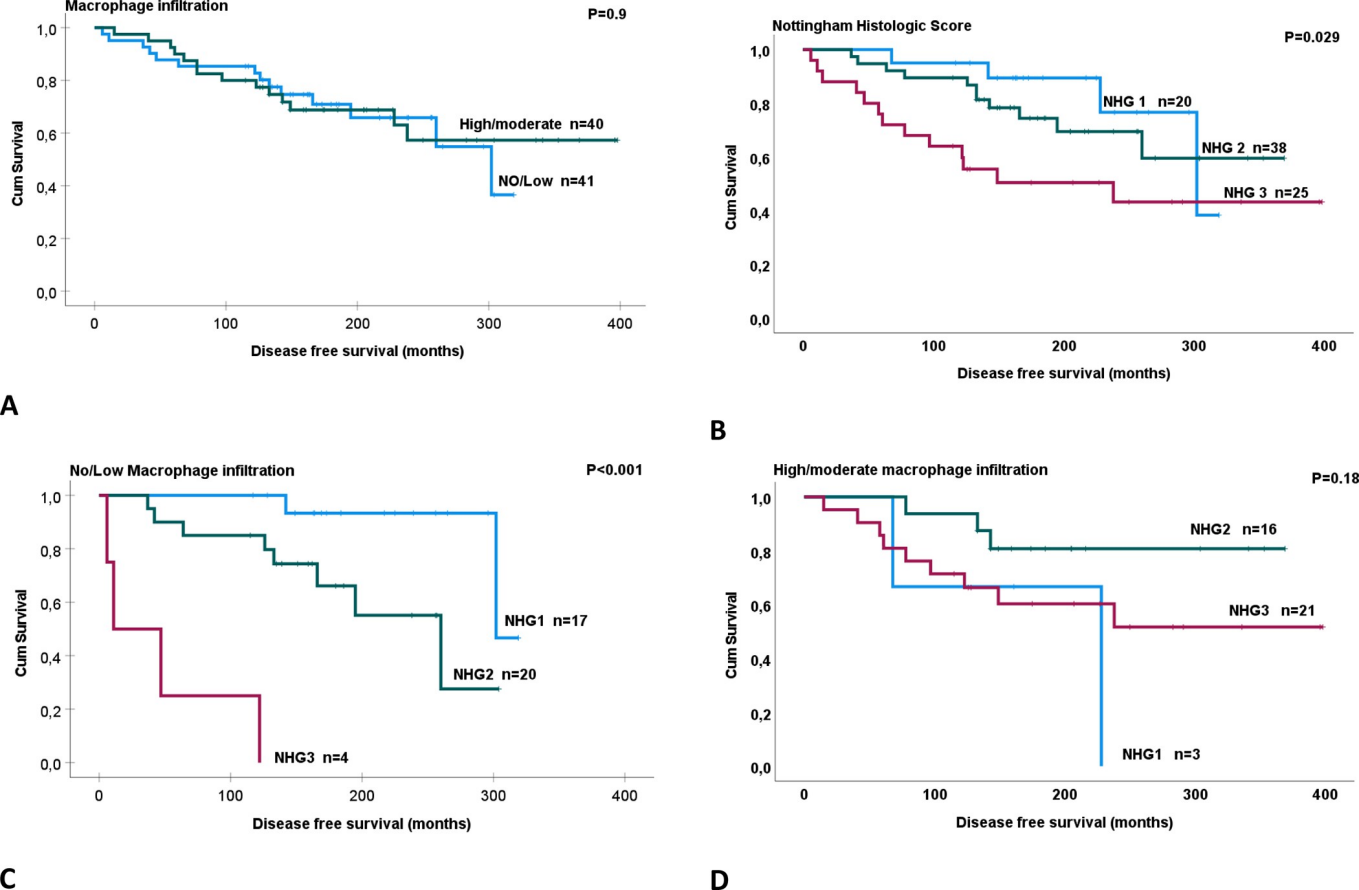
Kaplan-Meier curves demonstrating disease-free survival (DFS) in relation to (A) Nottingham Histologic Grade (NHG) and (B) macrophage infiltration for 83 patients with non-metastasized pT1-pT2 breast cancers treated with breast conserving surgery. In subgroup analysis, panels C and D show DFS in relation to NHG in patients having tumors with No/low and High/moderate macrophage infiltration, respectively. The comparison in this survival analysis is estimated according to the log-rank (Mantel-Cox) test.

Since tumors with moderate and high MI show similar differences in relation to ER and PR status, Ki-67 index, and NHG, we chose to merge these two groups into a new group named moderate/high. To explore the prognostic impact of MI and in relation to NHG, we initially examined the DFS in relation to each of these variables individually. There was no difference in DFS between patients with no/low (236 months) and moderate/high (283 months) MI (p = 0.9) (Fig 5A). As expected, DFS decreased proportionally to NHG per se with DFS rates of 278, 282, and 232 months for patients with NHG1, NHG2, and NHG3, respectively (P = 0.029) (Fig 5B).

Patients with no/low MI tumors also had reduced DFS in relation to NHG with DFS rates of 299, 211, and 45 months for NHG1, NHG2, and NHG3 tumors, respectively (p<0.001) (Fig 5C). Interestingly, we found the opposite trend of DFS in patients with moderate/high MI, although it was not statistically significant. DFS in the latter group was 175, 321, and 258 months for NHG1, NHG2, and NHG3 tumors, respectively (p = 0.18) (Fig 5D).

## Discussion

In this study, we investigate how the presence of M2 macrophages affects the hormonal status, GATA-3 expression, and differentiation states in selected patients with pT1-T2 BCs. We found that tumors with poorly differentiated states exhibit high MI and low GATA-3 expression. GATA-3 expression was associated with ER and PR expression but inversely correlated to MI. To our knowledge, this is the first study exploring the relationship between GATA-3, MI, and NHG in BC.

Tumors evolve by clonal selection of mutated cell populations with unconstrained proliferation and invasion ability. The mechanisms of invasive cancer cell selection and the geno- and phenotypes required for metastasis in tumor biology are still unclear. However, it is now widely accepted that tumor morphology and the dominance of the invasive phenotypes largely depend on selection by the host tissue microenvironment [1, 2, 17]. Macrophages are actively recruited by tumor cells, and once in the tumors, TAMs support an immunosuppressive microenvironment, promoting angiogenesis and the metastatic potential of cancer cells. For example, TAMs suppress anti-tumor immunity by inhibiting the programmed death-1 (PD-1) T cells and their ligands, PD-L1 and PD-L2 [18, 19]. In an *in vivo* mouse model, DeNardo et al. reported that inhibition of macrophage recruitment with CSF1 receptor antagonists in combination with chemotherapy (paclitaxel) reduced the growth of the primary BCs and pulmonary metastasis [20]. In a similar experimental study, Yang et al. show that the depletion of macrophages will reduce breast cancer stem cells and decreases tumorigenicity and metastasis of mammary tissue [21]. In addition to promoting tumor progression, macrophages promote endocrine resistance through, e.g., downregulation of ER and PR and activation of the PI3K/Akt/ mTOR pathway [22, 23]. In the current study, M2-specific MI is inversely correlated to decreased BC cell expression of ER- and PR and is proportional to tumor cell proliferation detected as Ki-67 index. Moreover, high MI was related to a significant decrease in GATA-3 expression and related to advanced tumor grade indicating that increased MI is associated with poorly differentiated states in BC. Together with comprehensive experimental observation [17, 18, 22–25], these findings suggest that the malignant potential of BC and probably its response to hormonal therapy does not depend solely on the biology of the tumor cells themselves. Hence, M2 macrophages are likely a crucial player in BC pathophysiology. From the clinical perspective, this is an important mechanism, as inhibition of MI may tentatively improve the prognostic assessment and potentiate the oncological treatment of BC.

In normal development, cell differentiation is a process by which proliferating cells gradually acquire tissue-specific function by changing phenotype. During carcinogenesis, tumor cells lose tissue-specific markers developing a de-differentiated state with increased proliferative capacity and plasticity. The morphology of BC cells is determined by the degree of their differentiation. However, the differentiation process in BC cells is not unidirectional towards differentiated luminal epithelial cells, as this lineage-specific differentiation is frequently altered or reversed [26, 27].

Comprehensive evidence indicates that GATA-3 is essential in the morphogenesis and regulation of mammary epithelial differentiation [28], constituting a valuable tissue-specific marker for confirming the epithelial or mesenchymal origin of tumors [28–30]. GATA-3 is associated with ER expression and a better response to hormonal treatment. However, the prognostic value of GATA-3 is still discussed [31–33].

Even if GATA-3 is associated with the differentiation of BC, few studies have explored the correlation between GATA-3 expression and BC differentiation. In all these studies, different approaches were used to evaluate positivity and determine cut-off rates for GATA-3 expression [25, 34–36]. Min et al. was the only group that used receiver operating characteristics to estimate the optimal cut-off rate (5% with an area under cover of 0.566) of GATA-3 [37]. These studies did not explore the extent of GATA-3 expression, i.e., the proportion of GATA-3 positive tumor cells in relation to the NHG. In this study, we avoided determining a cut-off rate nor estimating the prognostic value of GATA-3 as the primary endpoint because all BC tumor stages are not represented in this patient material (only pT1-2 tumors). Dichotomization is commonly used in clinical studies, but it is precarious to use this approach and split the continuous value of a marker when examining its correlation to other tumor variables [38]. Here we show that GATA-3 expression was proportional to ER and PR expression, consistent with observations reported in several previous studies [5, 7, 28, 31, 39]. GATA-3 expression was significantly lower in NHG3 compared to NHG1 and NHG2 tumors. Interestingly, there was no difference in GATA-3 expression between NHG1 and NHG2 tumors.

This study does not reveal the reason why NHG1 and NHG2 tumors exhibit a similar extent of GATA-3 expression. Tentatively, this similarity may be due to several factors, such as variations of GATA-3 expression in relation to individual assessment variables included in the NHG criteria (tubule formation, nuclear pleomorphism, and mitotic activity) or that de facto that NHG1 and NHG2 tumors show similar GATA-3 expression. For many years NHG has been an established BC scoring system and has proven to be an independent prognostic factor [40]. However, it is proposed that the mitotic index per se [41] better reflects BC malignant behaviour, as with the mitotic index is possible to divide the BC patients into groups with different prognoses [42, 43]. Hence, it should be logical that the expression of a biological marker, such as GATA-3, varies in relation to the assessment criteria included in the NHG classification. Further studies with larger patient samples and all BC tumor stages are needed to explore this topic.

As discussed previously, macrophages promote breast cancer progression, and that increased MI is related to advanced tumor stages and poor prognosis [12–14]. To the best of our knowledge, MI is not investigated in relation to GATA-3 expression nor its prognostic impact in association with NHG. In the current study, high MI was correlated to low GATA-3 expression and low differentiated states, NHG3. The MI itself was not related to the DSF. However, the DFS decreased inversely in relation to NHG in patients having tumors with no/low MI. Interestingly, no significant differences in DFS were found in relation to NHG in patients having tumors with moderate/high MI, indicating that MI influences the impact of NHG on DSF.

## Conclusion

This study provides further insight into the biological role and clinical significance of MI and GATA-3 in pT1-T2 BC. Macrophage infiltration is associated with differentiation states, hormonal status, and GATA-3 expression in BC. Moreover, the DFS analysed in relation to NHG is influenced by the extent of MI. These observations indicate that the extent of MI in the tumor microenvironment might impact the prognosis and treatment outcomes in BC, regardless of the morphological and hormonal states of the tumor cells.

## Supporting information

S1 Fig Flow chart showing the selection of 83 breast cancer patients treated with breast-conserving surgery. All patients had no lymph node or distant metastasis at the time of surgery, and the tumors were completely removed.
(DOCX)

## References

[1] Liotta L.A. and Kohn E.C. The microenvironment of the tumour-host interface. Nature, 2001. 411(6835): p. 375–9. 11357145 10.1038/35077241

[2] Hanahan D. and Weinberg R.A. The hallmarks of cancer. Cell, 2000. 100(1): p. 57–70. 10647931 10.1016/s0092-8674(00)81683-9

[3] Sørlie T., et al. Gene expression patterns of breast carcinomas distinguish tumor subclasses with clinical implications. Proceedings of the National Academy of Sciences, 2001. 98(19): p. 10869–10874. 10.1073/pnas.191367098 PMC58566 11553815

[4] Lowry J.A. and Atchley W.R. Molecular evolution of the GATA family of transcription factors: conservation within the DNA-binding domain. J Mol Evol, 2000. 50(2): p. 103–15. 10684344 10.1007/s002399910012

[5] Kouros-Mehr H., et al. GATA-3 maintains the differentiation of the luminal cell fate in the mammary gland. Cell, 2006. 127(5): p. 1041–55. 17129787 10.1016/j.cell.2006.09.048 PMC2646406

[6] Cancer Genome Atlas N. Comprehensive molecular portraits of human breast tumours. Nature, 2012. 490(7418): p. 61–70. 23000897 10.1038/nature11412 PMC3465532

[7] Parikh P., et al. GATA-3 expression as a predictor of hormone response in breast cancer. J Am Coll Surg, 2005. 200(5): p. 705–10. 15848360 10.1016/j.jamcollsurg.2004.12.025

[8] Lewis C.E. and Pollard J.W. Distinct role of macrophages in different tumor microenvironments. Cancer Res, 2006. 66(2): p. 605–12. 16423985 10.1158/0008-5472.CAN-05-4005

[9] Condeelis J. and Pollard J.W. Macrophages: obligate partners for tumor cell migration, invasion, and metastasis. Cell, 2006. 124(2): p. 263–6. 16439202 10.1016/j.cell.2006.01.007

[10] Lin E.Y. and Pollard J.W. Tumor-associated macrophages press the angiogenic switch in breast cancer. Cancer Res, 2007. 67(11): p. 5064–6. 17545580 10.1158/0008-5472.CAN-07-0912

[11] Pollard J.W. Tumour-educated macrophages promote tumour progression and metastasis. Nat Rev Cancer, 2004. 4(1): p. 71–8. 14708027 10.1038/nrc1256

[12] Volodko N., et al. Tumour-associated macrophages in breast cancer and their prognostic correlations. The Breast, 1998. 7(2): p. 99–105.

[13] Jeong H., et al. Tumor-associated macrophages as potential prognostic biomarkers of invasive breast cancer. Journal of breast cancer, 2019. 22(1): p. 38–51. 30941232 10.4048/jbc.2019.22.e5 PMC6438840

[14] Garvin S., et al. Differences in intra-tumoral macrophage infiltration and radiotherapy response among intrinsic subtypes in pT1-T2 breast cancers treated with breast-conserving surgery. Virchows Arch, 2019. 475(2): p. 151–162. 30915533 10.1007/s00428-019-02563-3 PMC6647441

[15] Fredriksson I., et al. Time trends in the results of breast conservation in 4694 women. Eur J Cancer, 2001. 37(12): p. 1537–44. 11506963 10.1016/s0959-8049(01)00168-x

[16] Cardoso F., et al. Early breast cancer: ESMO Clinical Practice Guidelines for diagnosis, treatment and follow-up. Ann Oncol, 2019. 30(10): p. 1674. 31236598 10.1093/annonc/mdz189

[17] Fidler I.J. The pathogenesis of cancer metastasis: the ’seed and soil’ hypothesis revisited. Nat Rev Cancer, 2003. 3(6): p. 453–8. doi: 10.1038/nrc1098 12778135

[18] Gordon S.R., et al. PD-1 expression by tumour-associated macrophages inhibits phagocytosis and tumour immunity. Nature, 2017. 545(7655): p. 495–499. 28514441 10.1038/nature22396 PMC5931375

[19] Spary L.K., et al. Tumor stroma-derived factors skew monocyte to dendritic cell differentiation toward a suppressive CD14(+) PD-L1(+) phenotype in prostate cancer. Oncoimmunology, 2014. 3(9): p. e955331. doi: 10.4161/21624011.2014.955331 25941611 PMC4292218

[20] DeNardo D.G., et al. Leukocyte complexity predicts breast cancer survival and functionally regulates response to chemotherapy. Cancer Discov, 2011. 1(1): p. 54–67. 22039576 10.1158/2159-8274.CD-10-0028 PMC3203524

[21] Yang J., et al. Tumor-associated macrophages regulate murine breast cancer stem cells through a novel paracrine EGFR/Stat3/Sox-2 signaling pathway. Stem Cells, 2013. 31(2): p. 248–58. 23169551 10.1002/stem.1281

[22] Li D., et al. Tumor-associated macrophages secrete CC-chemokine ligand 2 and induce tamoxifen resistance by activating PI3K/Akt/mTOR in breast cancer. Cancer Sci, 2020. 111(1): p. 47–58. 31710162 10.1111/cas.14230 PMC6942430

[23] Castellaro A.M., et al. Tumor-Associated Macrophages Induce Endocrine Therapy Resistance in ER+ Breast Cancer Cells. Cancers (Basel), 2019. 11(2). doi: 10.3390/cancers11020189 30736340 PMC6406935

[24] Riaz N., et al. Prognostic Significance of CSF-1R Expression in Early Invasive Breast Cancer. Cancers (Basel), 2021. 13(22). doi: 10.3390/cancers13225769 34830923 PMC8616299

[25] Hisamatsu Y., et al. Impact of GATA-3 and FOXA1 expression in patients with hormone receptor-positive/HER2-negative breast cancer. Breast Cancer, 2015. 22(5): p. 520–8. 24415069 10.1007/s12282-013-0515-x

[26] van Deurzen C.H., et al. Metaplastic breast carcinoma: tumour histogenesis or dedifferentiation? J Pathol, 2011. 224(4): p. 434–7. doi: 10.1002/path.2872 21462188

[27] Friedmann‐Morvinski D. and Verma I.M. Dedifferentiation and reprogramming: origins of cancer stem cells. EMBO reports, 2014. 15(3): p. 244–253. 24531722 10.1002/embr.201338254 PMC3989690

[28] Kouros-Mehr H., et al. GATA-3 links tumor differentiation and dissemination in a luminal breast cancer model. Cancer Cell, 2008. 13(2): p. 141–52. 18242514 10.1016/j.ccr.2008.01.011 PMC2262951

[29] Zheng R. and Blobel G.A. GATA Transcription Factors and Cancer. Genes & Cancer, 2010. 1(12): p. 1178–1188. 10.1177/1947601911404223 PMC3092280 21779441

[30] Kouros-Mehr H., et al. GATA-3 and the regulation of the mammary luminal cell fate. Curr Opin Cell Biol, 2008. 20(2): p. 164–70. 18358709 10.1016/j.ceb.2008.02.003 PMC2397451

[31] Voduc D., Cheang M., and Nielsen T. GATA-3 expression in breast cancer has a strong association with estrogen receptor but lacks independent prognostic value. Cancer Epidemiol Biomarkers Prev, 2008. 17(2): p. 365–73. 18268121 10.1158/1055-9965.EPI-06-1090

[32] Mehra R., et al. Identification of GATA3 as a breast cancer prognostic marker by global gene expression meta-analysis. Cancer Res, 2005. 65(24): p. 11259–64. 16357129 10.1158/0008-5472.CAN-05-2495

[33] Guo Y., et al. Prognostic and clinicopathological value of GATA binding protein 3 in breast cancer: A systematic review and meta-analysis. PLoS One, 2017. 12(4): p. e0174843. 28394898 10.1371/journal.pone.0174843 PMC5386271

[34] Albergaria A., et al. Expression of FOXA1 and GATA-3 in breast cancer: the prognostic significance in hormone receptor-negative tumours. Breast Cancer Res, 2009. 11(3): p. R40. 19549328 10.1186/bcr2327 PMC2716509

[35] Gulbahce H.E., et al. Significance of GATA-3 expression in outcomes of patients with breast cancer who received systemic chemotherapy and/or hormonal therapy and clinicopathologic features of GATA-3-positive tumors. Hum Pathol, 2013. 44(11): p. 2427–31. doi: 10.1016/j.humpath.2013.05.022 23998430

[36] Querzoli P., et al. GATA3 as an Adjunct Prognostic Factor in Breast Cancer Patients with Less Aggressive Disease: A Study with a Review of the Literature. Diagnostics (Basel), 2021. 11(4). doi: 10.3390/diagnostics11040604 33800667 PMC8066261

[37] Min K.W., et al. Negative association between GATA3 and fascin could predict relapse-free and overall survival in patients with breast cancer. Virchows Arch, 2016. 468(4): p. 409–16. 26719157 10.1007/s00428-015-1894-5

[38] Irwin J.R. and McClelland G.H. Negative Consequences of Dichotomizing Continuous Predictor Variables. Journal of Marketing Research, 2003. 40(3): p. 366–371.

[39] Hoch R.V., et al. GATA-3 is expressed in association with estrogen receptor in breast cancer. Int J Cancer, 1999. 84(2): p. 122–8. 10096242 10.1002/(sici)1097-0215(19990420)84:2<122::aid-ijc5>3.0.co;2-s

[40] Sundquist M., et al. Applying the Nottingham Prognostic Index to a Swedish breast cancer population. South East Swedish Breast Cancer Study Group. Breast Cancer Res Treat, 1999. 53(1): p. 1–8. 10206067 10.1023/a:1006052115874

[41] Baak J.P., et al. Prognostic indicators in breast cancer—morphometric methods. Histopathology, 1982. 6(3): p. 327–39. doi: 10.1111/j.1365-2559.1982.tb02727.x 7095760

[42] Lipponen P.K., Collan Y., and Eskelinen M.J. Volume corrected mitotic index (M/V index), mitotic activity index (MAI), and histological grading in breast cancer. Int Surg, 1991. 76(4): p. 245–9. 1663917

[43] Aloysius M.M., et al. Predictive value of tumor proliferative indices in periampullary cancers: Ki-67, mitotic activity index (MI) and volume corrected mitotic index (M/V) using tissue microarrays. World J Surg, 2010. 34(9): p. 2115–21. doi: 10.1007/s00268-010-0681-3 20556608

