# Supplementary material for: GATA-3 expression in breast cancer is related to intratumoral M2 macrophage infiltration and tumor differentiation
Source: PLoS One. 2023 Mar 30;18(3):e0283003. doi: 10.1371/journal.pone.0283003 (PMC10062580; doi:10.1371/journal.pone.0283003)
Supplement: S1 Fig — All patients had no lymph node or distant metastasis at the time of surgery, and the tumors were completely removed. (DOCX) [file pone.0283003.s001.docx]

**Supplement figure:** Chart flow showing the patient material and selection of study patient cohort and control group.

Age-matched women with breast cancer without local recurrence. Data from breast cancer registry of the southeastern region of Sweden between 1983-2008. N=1164

Women with breast cancer and local recurrence. Data from breast cancer registry of the southeastern region of Sweden between 1983-2008. N=1164

Total patient cohort of 2328 cases

Exclusion:

- Mastectomy N=668

- Re-resection N=48

- Wrong classification of LR N=14

- Not radical resection N=241

- Lymph node metastasis N=808

- Distant metastasis N=65

- Not ipsilateral LR N=20

- Missing data N=92

- Tumour blocks not available N=288

TMA created from primary tumors from eligible patients according to inclusion criteria N=84

Patients included in final analyses N=83

Excluded due to IHC technical failure N=1

BC without postoperative radiotherapy N=42

BC with postoperative radiotherapy N=41

Ipsilateral local recurrence N=23

No ipsilateral local recurrence N=25

No ipsilateral local recurrence N=19

Ipsilateral local recurrence N=16
